# Applications of Microalgal Biotechnology for Disease Control in Aquaculture

**DOI:** 10.3390/biology7020024

**Published:** 2018-04-12

**Authors:** Patai Charoonnart, Saul Purton, Vanvimon Saksmerprome

**Affiliations:** 1 Center of Excellence for Shrimp Molecular Biology and Biotechnology, Mahidol University, Bangkok 10400, Thailand; patai.cha@biotec.or.th; 2 National Center for Genetic Engineering and Biotechnology (BIOTEC) Thailand Science Park, Pathumthani 12120, Thailand; 3 Institute of Structural and Molecular Biology, University College London, London WC1E 6BT, UK; s.purton@ucl.ac.uk

**Keywords:** aquaculture, chloroplast transformation, disease control, microalgae, nuclear transformation, vaccine

## Abstract

Aquaculture industries, and in particular the farming of fish and crustaceans, are major contributors to the economy of many countries and an increasingly important component in global food supply. However, the severe impact of aquatic microbial diseases on production performance remains a challenge to these industries. This article considers the potential applications of microalgal technology in the control of such diseases. At the simplest level, microalgae offer health-promoting benefits as a nutritional supplement in feed meal because of their digestibility and high content of proteins, lipids and essential nutrients. Furthermore, some microalgal species possess natural anti-microbial compounds or contain biomolecules that can serve as immunostimulants. In addition, emerging genetic engineering technologies in microalgae offer the possibility of producing ‘functional feed additives’ in which novel and specific bioactives, such as fish growth hormones, anti-bacterials, subunit vaccines, and virus-targeted interfering RNAs, are components of the algal supplement. The evaluation of such technologies for farm applications is an important step in the future development of sustainable aquaculture.

## 1. Introduction

According to a 2016 report by the Food and Agriculture Organization of the United Nations (FAO), aquaculture marine food production has dramatically increased in the years since 1994, while capture fishery yields have remained relatively unchanged [1]. During this time, the aquaculture industry has become an important contributor to the national economies of many countries, in particular China, Mexico, and South-East Asian (SEA) countries. World aquaculture production in 2014 was valued up to US$160.2 billion, of which US$99.2 billion were from finfish, US$19 billion from molluscs, US$36.2 billion from crustaceans, and US$3.7 billion from other aquatic animals including amphibians. However, this important industry is constantly threatened by the possibility of widespread infection of the intensively farmed animals, which can result in dramatic losses [2]. For example, the major diseases causing high losses in shrimp stocks are from viral pathogens (the White Spot Syndrome Disease (WSSD) and the Yellow Head Disease (YHD)) and from bacterial pathogens (the Early Mortality Syndrome (EMS)) [3,4]. Diseases in fish are mostly caused by bacterial pathogens, the most prevalent of which are species of *Aeromonas*, *Pseudomonas*, and *Vibrio* [5]. More recently, a number of viral groups have also been reported as causative agents of disease in fish, including Betanodavirus such as nervous necrosis virus (NNV), Megalocyticvirus such as infectious spleen and kidney necrosis virus (ISKNV), and red sea bream iridovirus (RSIV) [6,7]. There is therefore a pressing need to find effective strategies to address these continuous and unpredictable diseases.

The management of infection using antibiotics or other chemotherapeutic agents that target bacterial pathogens and eukaryotic parasites is widespread within the aquaculture industry [8]. However, such a strategy does not afford protection against viral diseases, is potentially harmful to the environment, raises concerns regarding antibiotic contamination of the product, and encourages the emergence and spread of drug resistance amongst pathogens and parasites [9,10]. A better and safer strategy for disease control is vaccination, and a number of vaccines have been shown to be highly effective against specific viral or bacterial pathogens of fish [11]. Nevertheless, there are economic, technical, and regulatory challenges associated with vaccination. The cost of production and ‘cold-chain’ storage of vaccines (whether these are live-attenuated or killed whole microorganisms, recombinant subunit vaccines, or DNA vaccines) is often significantly higher than the production costs for antibiotics. The effective delivery of a vaccine dose to each animal also presents a challenge. Whilst intra-parenteral or intra-muscular delivery by injection minimizes vaccine wastage and ensures effective administration of a known amount, the process of capturing, handling, anesthetizing, and injecting the fish is very labor-intensive and costly and can cause injury to the fish. Furthermore, it is not feasible for small, juvenile fish or for crustaceans and impractical when repeat vaccinations are required. An alternative method involves immersion in a suspension of the vaccine using a bath, dip, or spray, but this can also be stressful to the fish and expensive in terms of amount of vaccine required.

The simplest and most sought after method is oral delivery of the vaccine by formulating it into the aquaculture feed. This approach allows mass vaccination of fish of all sizes, is non-stressful, requires little technical skill by the operator, and allows repeat dosing [11,12]. The key drawback of oral vaccination is the uncertainly and efficacy of a delivered dose, given that the vaccine can be lost through degradation—both in the water prior to feeding and in the fish gut through digestion prior to uptake by the immune tissue in the lower intestinal tract. As a consequence, oral vaccination often results in only a limited duration of immunity and may necessitate repeat dosing. Furthermore, variation in feeding between individual animals results in a Poisson distribution of the received dose. All of these factors mean that significantly more vaccine is required than for injection, and robust methods for DNA or antigen encapsulation are needed to allow efficient delivery to the immune tissue [13].

## 2. The Application of Microalgae in Aquaculture

Microalgae are a natural food source for many small aquaculture animals including larval shrimp and fish fry, and several microalgal species are used commercially as live prey or as dried whole algal feed [14]. The most frequently used species are those of *Chlorella*, *Tetraselmis*, *Isochrysis*, *Pavlova*, *Phaeodactylum*, *Chaetoceros*, *Nannochloropsis*, *Skeletonema*, and *Thalassiosira*, with a combination of different species often employed to provide the right balance of protein, lipids, and essential micronutrients [15]. In older animals, the supplement of fishmeal-based or grain-based feed with algal-derived biomass contributes additional nutritional value and also supplies necessary pigments such as the red astaxanthin that colours the flesh of salmonids and shellfish [15]. In addition to the inherent nutritional benefits, algal supplements have been reported to contain various compounds that serve as non-specific immunostimulants, improving the innate defense mechanisms in the animal and thereby providing enhanced resistance to pathogens [15]. Finally, many microalgal species have been reported to produce anti-microbial compounds that might be effective against particular bacterial, viral, and protist pathogens [16]. Microalgae, therefore, represent a beneficial component of finfish and shellfish feed, with these phototrophic microorganisms cultured in low-cost photobioreactors, either on-site (to provide live algae as a ‘green water’ supply) or off-site at a more appropriate location for algal cultivation (producing dried algae, frozen algal concentrates, or algal pastes suitable for storage and shipping). Moreover, the development of genetic engineering technologies for many algal species [17,18] offers the possibility of ‘functionalising’ the algal feed by engineering the algal cell to produce a novel bioactive, such as a vaccine, growth hormone, or anti-microbial agent. The cell then serves as a natural bio-encapsulation device for oral delivery of the bioactive to the target tissue. Of particular significance in this regard is the development of chloroplast engineering for several species. This offers both the possibility of high-level and stable accumulation of transgenic products in the organelle, but also an additional layer of bio-encapsulation provided by the multiple chloroplast membranes [19].

**Figure 1:**
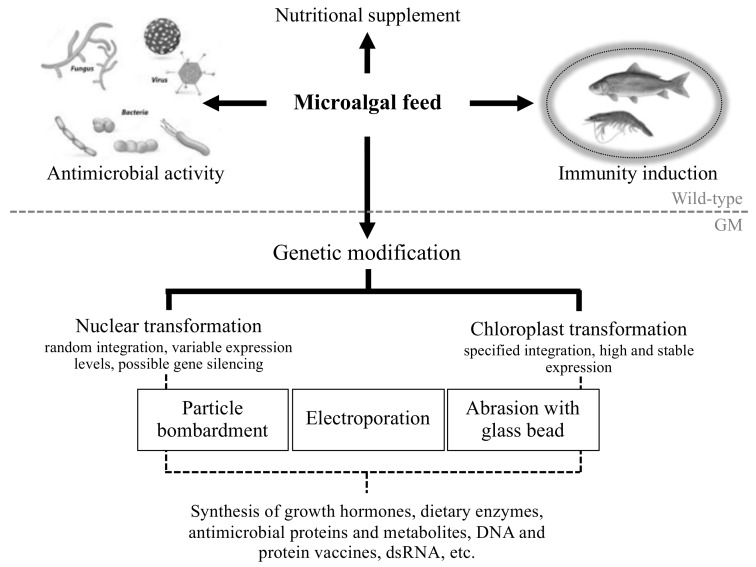
Overview of the current strategies for microalgal exploitation in aquaculture.

This review, therefore, focuses on the use of both natural and transgenic microalgal species as feed to control aquaculture diseases particularly in shrimp and fish, since these contribute a large proportion of total aquaculture production. The integration of microalgae into aquaculture is summarized in Figure 1.

## 3. Microalgae as a Health-Promoting Supplement for Coping with Aquaculture Disease

Bacteria are a major cause of aquatic animal diseases, and the number of antibiotic-resistant bacterial pathogens is continuously increasing. Various microalgal species exhibit natural anti-bacterial activities, and some also contain biomolecules that serve as immunostimulants [15,16]. Consequently, both crude algal extracts and whole cells have been used as a replacement or as a supplement in shrimp or fish feed to help address the issue of bacterial infections [20,21]. Members of the *Vibrio* bacterial genus are most often used as a target pathogen to test the inhibitory effect of microalgae. For example, a microalgal homogenate from *Tetraselmis suecica* showed good antibacterial activity in vitro against several *Vibrio* species including significant shrimp pathogens such as *Vibrio alginolyticus*, *Vibrio anguillarum, Vibrio parahaemolyticus*, and *Vibrio vulnificus* [22]. This study revealed a marked decrease in bacterial mobility with elongation and vacuolisation of the cells, which resulted in an inhibition of bacterial growth. Other microalgal species commonly used as aquaculture feed have similarly shown antibacterial activity in vitro against particular shrimp and fish pathogens. These algae include *Chaetoceros lauderi*, *Dunaliella tertiolecta*, *Euglena viridis*, *Phaeodactylum tricornutum* and *Stichochrysis immobilis* [16,23,24]. Natrah et al. (2011) reported that extracts from several marine and freshwater algae were able to interfere with quorum sensing mediated by the small molecule acyl-homoserine lactone in test bacteria including the pathogen *Vibrio harveyi*. This is significant, since quorum sensing is a mechanism of cell-to-cell communication that plays a key role in bacterial virulence [25]. Furthermore, several studies have shown that algal-derived long-chain polyunsaturated fatty acids (LC-PUFAs) such as eicosapentaenoic acid (EPA), as well as algal sterols, have anti-bacterial properties and can be effective against both Gram-positive and Gram-negative bacteria [26,27]. Therefore, inclusion of microalgae in the diet of aquatic animals is likely to contribute to a reduced risk of bacterial infection.

Microalgal feeding also appears to improve disease tolerance of the host animal. Replacement of fish oil with algal meal containing high amounts of the LC-PUFAs docosahexaenoic acid (DHA) and arachidonic acid (AA), significantly improved immune parameters such as total haemocyte count, phenoloxidase activity, superoxide dismutase activity, and bactericidal activity in the post-larval stage of the Pacific white shrimp (*Litopenaeus vannamei*), resulting in improved survival rates against *V. harveyi* infection [28]. Our own unpublished studies into immunity induction following replacement of either fish meal or fish oil with microalgae revealed a proportion-dependent effect in agreement with Nonwachai et al. [28]. The carotenoid pigments lutein, zeaxanthin, and astaxanthin that are abundant in microalgae were also reported to increase the survival of shrimp and fish, as well as of other crustaceans, when subjected to disease infection [29,30]. Vitamin C, which is found in high amounts in several microalgal species, was reported to boost immunity in shrimp resulting in decreased mortality from *Vibrio* species [31,32]. Radhakrishnan et al. found that post larvae of the giant freshwater prawn *Macrobrachium rosenbergii* had significantly higher vitamin C levels following a feed diet containing cyanobacteria (*Spirulina platensis*), green alga (*Chlorella vulgaris*), and fern (*Azolla pinnata*) [33]. However, there have been no studies confirming a relationship between higher antioxidant levels as a result of microalgal feeding and disease tolerance. Nevertheless, several studies have reported the use of a feed diet containing microalgae to combat bacterial pathogen in aquaculture and attributed this to a combination of antibacterial activity and host immunity induction by microalgal-derived compounds [34,35,36].

Immunostimulants also play a significant role in terms of enhancing the protection against viral pathogens [36,37]. Feed diets supplemented with 80 mg/kg astaxanthin, present in high amounts in several green algal species such as *Haematococcus pluvialis* and usually used for improving coloration, conferred partial white spot syndrome virus (WSSV) protection and immunity and antioxidant enzyme induction in *L. vannamei* [38]. Similarly, *Dunaliella salina* was found most applicable for viral protection, in particular against WSSV, since this species contains high amounts of beta-carotene. A feed diet containing beta-carotene from *D. salina* at 15 mg/kg for six weeks had no significant effect on haemocyte count and phenoloxidase activity in black tiger shrimp (*Penaeus monodon*). However, a WSSV challenge test revealed a two–three-fold higher protection efficiency compared to normal feed [39]. *P. monodon* fed a diet supplemented with dry *D. salina* showed higher levels of prophenoloxidase than when fed a diet without *D. salina* and short-term induction of antioxidant substances such as superoxide dismutase and catalase. While *P. monodon* challenged with WSSV and fed a diet without *D. salina* showed 100% mortality within 48 h, an experimental group receiving a *D. salina*-containing diet showed a 60% survival on day 5 and, 100% mortality was delayed until day 10 [40]. Despite these promising results with crustaceans, we have not found any similar studies reporting the use of microalgae as feed for improved viral protection in fish.

## 4. Engineering Microalgae to Produce Novel Antiviral and Antibacterial Biomolecules

Recent advances in the genetic engineering of a wide range of microalgal species [17,18] are now making possible the development of bespoke strains for oral delivery of novel anti-microbial agents, as well as of other beneficial recombinant molecules, including growth promoters and dietary enzymes such as phytases and cellulases [41,42]. For some species—in particular, the green alga *Chlamydomonas reinhardtii*—advanced synthetic biology methods are being developed for engineering both the nucleus and the chloroplast genomes [43,44]. Successful introduction of a functional transgene into a cell involves three requirements. The first is the physical delivery of the foreign DNA into the nuclear or chloroplast genome (i.e., ‘transformation’). In the case of microalgae, the small cell size and thick cell wall often present significant transformation challenges. However, techniques such as microparticle bombardment, electroporation, agitation with glass beads or silicon carbide whiskers, or *Agrobacterium*-mediated DNA transfer, have all been exploited to address these challenges in different species [18,41]. Furthermore, the use of cell wall-deficient mutants or of algae at a natural stage in their life cycle characterized by a minimal cell wall has facilitated DNA delivery [45,46]. Integration of the DNA into the chloroplast genome can be directed to a specific locus via a process of homologous recombination, whereas integration into the nucleus of most algal species is uncontrolled, occurring essentially at random loci [18]. However, the ongoing development of genome targeting technologies for microalgae based on CRISPR-Cas, should allow precise and predictive insertion of transgenes into chosen loci in the nucleus [47]. This will avoid the issue of ‘position effects’ whereby the site of insertion can have a profound influence on the level and stability of transgene expression. The second requirement is the selection for the rare transformant lines within a large population of untransformed cells. Typically, selectable markers based on bacterial antibiotic-resistance genes have been used for both nuclear transformation (e.g., zeomycin or kanamycin resistance) or chloroplast transformation (e.g., spectinomycin or erythromycin resistance) [18]. However, the dispersal of algal material containing these genes into the aquatic environment is not desirable, given the possibility of horizontal transfer of the genes to local bacterial species. Consequently, selection strategies have been developed on the basis of the rescue of auxotrophic or phototrophic mutants with the corresponding wild-type algal gene, or the acquisition of a resistance phenotype conferred by a dominant mutation in the algal gene [18,48]. The final requirement is the successful expression of the introduced transgene within the algal cell. Typically, this involves optimization of the coding sequence to match the codon preference of the target genome (nuclear or chloroplast) in the chosen algal species, and fusion of this coding sequence to a promoter and to 5′ and 3′ untranslated regions (UTRs) known to be functional in the alga. Such *cis* elements are usually derived from endogenous genes such as those encoding components of the photosynthetic apparatus that are known to be highly expressed in the alga. However, plant and plant viral promoters (e.g., from maize and from Cauliflower Mosaic Virus) have also been shown to drive transgene expression in the nucleus [18,49], and synthetic promoters and 5′ UTRs that circumvent natural feedback regulations in the nucleus and chloroplast are being developed as orthogonal elements for transgenesis [50,51].

To date, progress in the development of transgenic microalgae as platforms for oral delivery of biomolecules for aquaculture disease is limited but encouraging. As detailed in Table 1, there are reports on the production of viral antigens, antimicrobial peptides, and double-stranded RNA (dsRNA) by nuclear and chloroplast transformations of several algal species. In one study, the feeding of Medaka fish with *Nannochloropsis oculata* expressing bovine lactoferricin—a broad-spectrum antimicrobial peptide that can kill or inactivate many bacterial, viral, and fungal pathogens—six hours prior to *V. parahaemolyticus* challenge showed a 70–85% survival rate compared to 5% survival rates in fish fed with the wild-type alga [52]. In another study, Feng et al. expressed the gene for viral envelope protein 28 (VP28) from WSSV in the nucleus of *D. salina* under the control of the maize *Ubi1* promoter and obtained 78 µg of recombinant protein per 100 ml of culture [53]. A cell lysate from the transgenic alga was used to coat commercial crayfish feed, and the animals were challenged with WSSV following feeding. Whilst the control challenge experiments resulted in 100% mortality, the VP28-fed crayfish showed significantly higher survival rates, with only 59% mortality. In a similar study, recombinant VP28 was produced by chloroplast transformation of *C. reinhardtii*, and the alga was fed to shrimp (*L. vannamei*) followed by challenge with WSSV. A significant rate of survival (87%) was found in shrimp receiving the transgenic *C. reinhardtii*, whereas no shrimp survived in the group fed the wild-type alga ([54], T.W. Brocklehurst, personal communication). Engineering of *C. reinhardtii* was also used by Siripornadulsil et al. [55] to produce the p57 antigen of *Renibacterium salmoninarum*, the causative agent of bacterial kidney disease (BKD). The researchers used two strategies: (1) chloroplast transformation to produce the full-length p57 protein that accumulated in the organelle; (2) nuclear transformation to produce a chimeric protein consisting of a 14-residue antigenic domain of p57 fused to a plasma membrane protein such that the p57 antigen would be presented on the algal cell surface. Juvenile rainbow trout fed a diet containing the dried algae or immersed in water containing the live algae were shown to produce antibodies to the p57 antigen.

Whilst these and other transgenic approaches given in Table 1 focus on the production of recombinant proteins, the production of recombinant double-stranded RNA (dsRNA) represents a promising alternative strategy for protection against an infecting virus [60,61]. Here, the dsRNA serves to trigger an RNA interference (RNAi) response in the animal that compromises the expression of target viral genes. Earlier work involving oral administration of *Escherichia coli* expressing dsRNA targeting shrimp viral genes resulted in a significant decrease in the viral copy number [61,62]. Subsequently, the successful integration of a hairpin DNA cassette expressing dsRNA targeting the yellow head virus (YHV) in the *C. reinhardtii* nucleus has been reported [59]. A challenge test after feeding *L. vannamei* shrimp with this transgenic alga demonstrated partial protection against the virus. It is likely that the lack of full protection was in part due to the degradation of the dsRNA by RNase III in the microalgal nucleus and that production in the chloroplast might be more effective because of the lack of such dsRNA processing in this organelle [63].

Indeed, the use of the algal chloroplast as a platform for producing therapeutic biomolecules is attractive for a number of reasons [44]. Firstly, as noted above, insertion of transgenes into the chloroplast genomes is precise and predictable, with the transgene targeted to a chosen locus. In contrast, nuclear transformation of most microalgal species results in unpredictable expression due to the position effects associated with the random integration of the transgene and to the uncontrolled integration of multiple transgene copies [64]. Secondly, there are no gene silencing mechanisms in the chloroplast, unlike in the nucleus; therefore, transgene expression is stable and does not require the maintenance of a selective pressure to ensure active expression. Thirdly, much higher levels of transgene expression can be achieved in the chloroplast, partly due to the high ploidy of the chloroplast genome and partly due to the use of promoter and UTRs from very highly expressed chloroplast genes. Finally, chloroplast transformation of *C. reinhardtii* can be achieved on the basis of the rescue of photosynthetic mutants carrying deletions in essential chloroplast genes such as *psbA* or *psbH.* This allows the generation of transgenic lines free of any antibiotic resistance markers [48,65]. We are currently exploiting this technology to produce marker-free chloroplast transformants of *C. reinhardtii* expressing dsRNA from convergent promoters. Our first transformant line accumulates dsRNA from the RdRp gene of YHV and is under development as an antiviral shrimp feed against YHV [66].

## 5. Challenges and Constraints in Algal Research

### 5.1. Further Advances in Microalgal Genetic Engineering

Significant advances have been made over the last ten years towards the development of genetic engineering technologies for microalgae, and there are reports of successful transformation of over 30 species [18]. However, further advances are needed, particularly for those species listed in Section 2 that are already established as a safe, palatable, easily digestible, and nutritious ‘whole-cell’ feed. A standardised ‘synthetic biology’ method of rapid DNA assembly using validated genetic parts need to be combined with efficient methods of DNA delivery into the algal genome in order to accelerate the process of designing and evaluating bespoke algal strains [67]. Furthermore, there is a need to address concerns surrounding the use of bacterial antibiotic resistance genes as selectable markers by developing markers based on endogenous algal genes. For those species for which the isolation of auxotrophic mutants is possible (i.e., those with a haploid genome or those with an established sexual cycle allowing the generation of homozygous recessive lines), the corresponding wild-type gene can be used for selection. Conversely, endogenous genes carrying a dominant mutation can be used as markers to transform wild-type cells, irrespective of their ploidy [68]. There is also a need to establish generic techniques for targeted insertion of transgenes into the microalgal nuclear genome based on the emerging CRISPR-Cas technology [48]. This would allow predictive and reproducible insertion of transgenes into loci identified as ‘safe harbours’ for stable, high-level expression [18]. Encouraging progress in this regard has been made by several groups who have developed facile CRISPR-Cas techniques for green algae and diatoms [69,70]. Alternatively, integration of transgenes into the nuclear chromosomes can be circumvented completely by the maintenance of the genes on stable episomal plasmids, as has been demonstrated for diatoms [70,71]. Other issues include: (1) the need to develop more advanced algorithms for codon optimization of synthetic transgenes for each particular microalgal species to ensure maximal translational efficiency [72,73]; (2) the need to develop promoter systems that are easily inducible in an industrial cultivation process [74,75]. Currently, most algal transgenics involves the use of constitutive promoters such that the recombinant product is synthesized during algal biomass production. This can negatively impact growth, either because of the extra metabolic burden or because of a toxic effect on the cell, and a better strategy is to induce product synthesis towards the end of the growth phase. Tightly regulated promoters with a large dynamic range, together with effective codon optimisation, would therefore help to achieve the highest cellular yield of the desired product.

Finally, there is a pressing need to develop chloroplast transformation methods for the key microalgal species for the many reasons discussed above. *C. reinhardtii* is the only microalgal species for which chloroplast transformation is currently well-established, although there are isolated reports of transformation of several other species including *D. tertiolecta* and *P. tricornutum* [44]. Nevertheless, there have been several reports that highlighted the potential of using chloroplast engineering to create bespoke strains as functional feed additives. For example, Stoffels et al. [76] produced highly active anti-bacterial enzymes in the chloroplast. These lytic enzymes (termed ‘endolysins’) are derived from bacteriophages and are highly specific for the bacterial host of the phage, in this particular case, *Streptococcus pneumoniae*. It is therefore feasible that similar microalgal strains could be created that produce one or more endolysins targeting bacterial pathogens of shrimp or fish [77]. Oral delivery of the algae could help protect the animal against the pathogens without affecting the natural microbiome. In a separate report, the same group showed that active human growth hormone could be produced in the *C. reinhardtii* chloroplast [78]. One could therefore imagine adding fish growth hormone (fGH) to the suite of recombinant products produced in the algal feed in order to boost fish growth. A precedent for oral delivery of fGH was established by two separate studies [78,79] in which tilapia larvae were fed with *N. oculata* engineered to produced fGH [78], and flounder fry were fed on similarly engineered *Chlorella ellipsoidea* [79]. In both cases, there was a significant increase in body weight when compared to control feedings with the non-transformed alga. Further precedents for oral delivery of other bioactives such as antiviral agents can be found in studies using alternative platforms, such as edible plants or microbes. This work can form the basis of future studies in which the agents are produced in the microalgal chloroplast. For example, feeding of rock bream fish with transgenic rice callus producing the major capsid protein of Iridovirus induced antibodies and protected the fish against the virus [80]. In a separate study, oral delivery of yeast expressing the viral capsid protein of infectious pancreatic necrosis virus induced a specific immune response and a reduced viral load in rainbow trout [81].

### 5.2. Large-Scale Production and Downstream Processing

A significant challenge in the commercial exploitation of algae for aquaculture is efficient large-scale production at a sufficiently low cost. Natural strains of algae can be cultivated outdoors in open ponds or in closed systems such as tubular or flat-panel photobioreactors, although biomass production costs are still high for an animal feed ingredient—in the range of 5–13 $/kg [82,83]. For production of genetically engineered algae, stricter regulations would require indoor cultivation under artificial lighting in a closed and contained system, thereby adding significantly to the costs. One practical solution is to reduce the capital costs (and the costs of skilled staff) by using a simple “hanging bag” system in which the algae is grown in sterile, single-use polythene tubing [18]. Each bag typically contains 30–40 L of culture and is mixed and carbonated through bubbling with an air/CO_2_ mix. With such a modular system, scaling is straightforward, as is the management of contamination within individual bags. Harvesting of the microalgae from the bulk medium is also a challenge given that the algal biomass represents only a few percent of the volume. Centrifugation or microfiltration methods are expensive and generally impractical, and a better strategy is to first concentrate the biomass by flocculation. A suitable and cheap flocculant is chitosan, since it is a natural byproduct of the crab industry, is already approved as a food additive, and is highly effective as a microalgal flocculant [84]. Finally, preservation of the algal biomass can be achieved either by freeze-drying (i.e., cryopreservation) or spray-drying [85]. Numerous studies have shown that recombinant therapeutic proteins produced in microalgae retain bioactivity following freeze-drying, and several studies have reported that the activity can remain stable for many months when the dried algal powder is stored at room temperature [44]. Drying, therefore, offers a simple method of bio-encapsulation and storage that avoids the need for a cold chain. However, this would need to be confirmed and optimised for each recombinant product [86], and it is noteworthy that there is one report where an algal vaccine was effective in eliciting an immune response in rabbit only when administered as a live feed, not as a dried feed [52].

### 5.3. Regulation, Risk, and Public Acceptance

Although this article highlights the potential of using microalgae to control aquaculture disease, it is important to consider also the issues and risks associated with such technology. Firstly, the commercial cultivation of an algal species may be subject to country-specific regulations if the species is considered non-native. Two considerations arise: (i) the import of a species might be regulated by the Nagoya Protocol on ownership and sharing of biological resources [87] if it has been isolated recently from another country; (ii) cultivation of an alien species might pose a significant ecological risk through accidental escape into the environment, and therefore should be subjected to appropriate risk assessment and approval [86]. For genetically modified microalgae, additional regulations and risk assessments would clearly apply [88,89]. Once cultivated, the algal biomass then needs to be treated to ensure that no viable cells survive prior to its formulation into feed and deliberate release into the aquatic environment. Drying might be sufficient to kill all the algal cells, however it is important to appreciate that certain species are able to form highly resistant spores under conditions of stress (e.g., *H. pluvialis*) or form similarly resistant zygospores as a result of homothallic mating (e.g., *Chlamydomonas monoica*). Such spores could survive the formulation process and then germinate when exposed to the nutrient-rich conditions of the fish ponds. A further consideration in this regard is the observation that some algal species appear able to serve as viral vectors, spreading a virus amongst the animal population. In one report, the spread of white spot syndrome virus amongst kuruma shrimp (*Marsupenaeus japonicus*) by various microalgal species was demonstrated, including species such as *Chorella* sp. and *D. salina* species that are already used in aquaculture feeds [90]. Finally, the horizontal transfer of transgenes from the algal biomass to environmental microorganisms (even if the algae are killed and dried, the DNA carrying a transgene can remain intact) represents a further risk, particularly if the algal material contains a marker gene based on bacterial antibiotic-resistance [91]. It is therefore vital that genetic engineers incorporate biocontainment strategies into transgenic lines, both at the organism level, by creating mutant strains that would fail to grow or compete in the natural environment (e.g., auxotrophic or mating-defective mutations [88]), and at the transgene level, by avoiding antibiotic-based selectable markers and incorporating functional safeguards into the transgene itself (e.g., the use of an orthogonal stop codon/tRNA system that allows the functioning of the gene only in the algal host [92]).

Public acceptance of products from aquaculture animals that have been reared using genetically modified (GM) feed ingredients is also a significant consideration. This is especially relevant if the product is being marketed as ‘healthy’ and ‘sustainable’, in which case, a “GM-free” certification might have more value to the company than the benefits afforded by a GM-based functional feed. In the same way that the meat industry is faced with the issue of public acceptance of animal feed derived from GM crops [93], the aquaculture industry will have to gauge the risks and benefits of adopting GM algae. Nevertheless, we are starting to see the emergence of algal biotechnology companies exploring the ‘functionalised algae’ space. Two examples are the Israeli company Transalgae Ltd. (Rehovot, Israel) and the Irish company MicroSynbiotiX Ltd. (Cork City, Ireland), both of which are developing algal-based oral vaccines for aquaculture. 

## 6. Conclusions

Microalgae have been shown to have potential to improve aquaculture production. The nutritional benefits of using microalgae as a source of proteins, lipids, and essential micronutrients in the feed are proven and are now being applied at the farm scale. Microalgae also offer opportunities for natural protection against microbial pathogens and, hence, disease prevention through the production of natural anti-microbial compounds and immunostimulants. Given that there are more than 10,000 species of freshwater and marine microalgae identified to date, but only a tiny percentage have been screened for such compounds, there is clearly a rich untapped resource for such bioactives. Furthermore, the rapidly developing field of algal genetic engineering and synthetic biology opens the door to designer strains that can serve as functional feed additives, supplying both natural nutrition and a suite of beneficial recombinants for both disease prevention and improved growth. Responsible adoption of this emerging biotechnology will help contribute to a sustainable aquaculture industry and the promotion of global food security.

## Figures and Tables

**Table 1:** Genetic engineering of microalgae to produce therapeutic proteins and biomolecules against aquaculture diseases.

Species	Site of Transgene Insertion	DNA Delivery Method and Selection System	Introduced Gene Product	Yield	Evidence for Functionality	Reference
*Chlamydomonas reinhardtii*	Chloroplast	Microparticle bombardment; restoration of photosynthesis	p57 secreted protein from *Renibacterium salmoninarum*, the cause of bacterial kidney disease (BKD) in salmonid fish	N.D. ^1^	Induction of anti-p57 antibodies in the blood of fish fed with the dried algae	[55]
		Microparticle bombardment; spectinomycin resistance	Viral envelope protein 28 (VP28) of white spot syndrome virus (WSSV) G-protein from infectious hematopoietic necrosis virus (IHNV) VP2 protein of infectious pancreatic necrosis virus (IPNV) p57 BKD antigen	0.2–21% TCP ^2^ <0.5% TCP <0.3% TCP <0.5% TCP	N.D. N.D. N.D. N.D.	[56]
		Microparticle bombardment; spectinomycin resistance	VapA and AcrV antigens of fish bacterial pathogen *Aeromonas salmonicida*	0.3% TSP ^3^ (VapA) 0.8% TSP (AcrV)	Initial fish feeding trials revealed no adverse effects of feeding, but also no immunological response to either antigen or protection against the pathogen (see: [57])	[58]
		Agitation with glass beads; restoration of photosynthesis	VP28 of white spot syndrome virus (WSSV)	N.D.	Challenge trials showed reduced mortality from WSSV for shrimp fed with algal meal containing the VP28 antigen	[54]
*Chlamydomonas reinhardtii*	Nucleus	Electroporation; rescue of arginine prototroph	14 amino acid antigenic domain of p57 fused to an endogenous plasma membrane protein	N.D.	Induction of anti-p57 antibodies in the blood of fish fed with the dried algae	[55]
		Agitation with glass beads; paromomycin resistance	374 bp double-stranded RNA targeting RdRp gene of yellow head virus (YHV)	45 ng ds-RNA per 100 mL culture	Challenge trials showed 22% improvement in survival rate against YHV for shrimp fed with algal meal containing ds-RNA	[59]
*Nannochloropsis oculata*	Nucleus	Electroporation, fluorescence of DsRed ^4^ reporter	Broad spectrum antimicrobial peptide Bovine Lactoferricin (LFB) fused to DsRed	N.D.	Medaka fish fed with algal meal containing LFB showed ~85% survival against *Vibrio parahymolyticus* infection compared to control (~5%)	[52]
*Dunaliella salina*	Nucleus	Agitating with glass beads; phosphinothricin resistance	VP28 of white spot syndrome virus (WSSV)	78 µg/100 mL culture	Challenge trials showed 41% survival rate of crayfish against WSSV	[53]

^1^ N.D.: Not Determined, ^2^ TCP: Total Cell Protein, ^3^ TSP: Total Soluble Protein, ^4^ DsRed: Red Fluorescence Protein.

## References

[1] The Food and Agriculture Organization of the United Nations (FAO) (2016). The State of World Fisheries and Aquaculture 2016. Contributing to Food Security and Nutrition for All.

[2] Bondad-Reantaso M.G., Subasinghe R.P., Arthur J.R., Ogawa K., Chinabut S., Adlard R., Tan Z., Shariff M. (2005). Disease and health management in Asian aquaculture. Vet. Parasitol..

[3] Kusumaningrum H.P., Zainuri M. (2015). Detection of bacteria and fungi associated with *Penaeus monodon* postlarvae mortality. Procedia Environ. Sci..

[4] Thitamadee S., Prachumwat A., Srisala J., Jaroenlak P., Salachan P.V., Sritunyalucksana K., Flegel T.W., Itsathitphaisarn O. (2016). Review of current disease threats for cultivated penaeid shrimp in Asia. Aquaculture.

[5] Austin B., Austin D.A. (2012). Bacterial Fish Pathogens; Disease of Farmed and Wild Fish.

[6] Crane M., Hyatt A. (2011). Viruses of fish: An overview of significant pathogens. Viruses.

[7] Subramaniam K., Shariff M., Omar A.R., Hair-Bejo M. (2012). Megalocytivirus infection in fish. Rev. Aquac..

[8] Defoirdt T., Sorgeloos P., Bossier P. (2011). Alternatives to antibiotics for the control of bacterial disease in aquaculture. Curr. Opin. Microbiol..

[9] Cabello F.C., Godfrey H.P., Buschmann A.H., Dölz H.J. (2016). Aquaculture as yet another environmental gateway to the development and globalisation of antimicrobial resistance. Lancet Infect. Dis..

[10] Watts J.E.M., Schreier H.J., Lanska L., Hale M.S. (2017). The rising tide of antimicrobial Resistance in aquaculture: Sources, sinks and solutions. Mar. Drugs.

[11] Plant K.P., Lapatra S.E. (2011). Advances in fish vaccine delivery. Dev. Comp. Immunol..

[12] Dhar A., Allnutt F. (2011). Challenges and opportunities in developing oral vaccines against viral diseases of fish. J. Mar. Sci. Res. Dev. S.

[13] Kibenge F.S., Godoy M.G., Fast M., Workenhe S., Kibenge M.J. (2012). Countermeasures against viral diseases of farmed fish. Antivir. Res..

[14] Spolaore P., Joannis-Cassa C., Duran E., Isambert A. (2006). Commercial applications of microalgae. J. Biosci. Bioeng..

[15] Shah M.R., Lutzu G.A., Alam A., Sarker P., Chowdhury M.A.K., Parsaeimehr A., Liang Y., Daroch M. (2018). Microalgae in aquafeeds for a sustainable aquaculture industry. J. Appl. Phycol..

[16] Falaise C., François C., Travers M.-A., Morga B., Haure J., Tremblay R., Turcotte F., Pasetto P., Gastineau R., Hardivillier Y. (2016). Antimicrobial compounds from Eukaryotic Microalgae against human pathogens and diseases in aquaculture. Mar. Drugs.

[17] Qin S., Lin H., Jiang P. (2012). Advances in genetic engineering of marine algae. Biotechnol. Adv..

[18] Spicer A., Purton S., Slocombe S.P., Benemann J.R. (2016). Genetic engineering of microalgae: Current status and future prospects. Microalgal Production for Biomass and High-Value Products.

[19] Taunt H.N., Stoffels L., Purton S. (2018). Green biologics: The algal chloroplast as a platform for making biopharmaceuticals. Bioengineered.

[20] Dang V.T., Li Y., Speck P., Benkendorff K. (2011). Effects of micro and macroalgal diet supplementations on growth and immunity of greenlip abalone, *Haliotis laevigata*. Aquaculture.

[21] Yaakob Z., Ali E., Zainal A., Mohamad M., Takriff M.S. (2014). An overview: Biomolecules from microalgae for animal feed and aquaculture. J. Biol. Res.-Thessalon..

[22] Austin B., Day J. (1990). Inhibition of prawn pathogenic *Vibrio* spp. by a commercial spray-dried preparation of *Tetraselmis suecica*. Aquaculture.

[23] Viso A., Pesando D., Baby C. (1987). Antibacterial and antifungal properties of some marine diatoms in culture. Bot. Mar..

[24] Das B., Pradhan J. (2010). Antibacterial properties of selected freshwater microalgae against pathogenic bacteria. Indian J. Fish..

[25] Natrah F., Kenmegne M.M., Wiyoto W., Sorgeloos P., Bossier P., Defoirdt T. (2011). Effects of micro-algae commonly used in aquaculture on acyl-homoserine lactone quorum sensing. Aquaculture.

[26] Benkendorff K., Davis A.R., Rogers C.N., Bremner J.B. (2005). Free fatty acids and sterols in the benthic spawn of aquatic molluscs, and their associated antimicrobial properties. J. Exp. Mar. Biol. Ecol..

[27] Desbois A.P., Mearns-Spragg A., Smith V.J. (2009). A fatty acid from the diatom *Phaeodactylum tricornutum* is antibacterial against diverse bacteria including multi-resistant *Staphylococcus aureus* (MRSA). Mar. Biotechnol..

[28] Nonwachai T., Purivirojkul W., Limsuwan C., Chuchird N., Velasco M., Dhar A.K. (2010). Growth, nonspecific immune characteristics, and survival upon challenge with *Vibrio harveyi* in Pacific white shrimp (*Litopenaeus vannamei*) raised on diets containing algal meal. Fish Shellfish Immunol..

[29] Merchie G., Kontara E., Lavens P., Robles R., Kurmaly K., Sorgeloos P. (1998). Effect of vitamin C and astaxanthin on stress and disease resistance of postlarval tiger shrimp, *Penaeus monodon* (Fabricius). Aquac. Res..

[30] Babin A., Biard C., Moret Y. (2010). Dietary supplementation with carotenoids improves immunity without increasing its cost in a crustacean. Am. Nat..

[31] Kontara E., Merchie G., Lavens P., Robles R., Nelis H., De Leenheer A., Sorgeloos P. (1997). Improved production of postlarval white shrimp through supplementation of L-ascorbyl-2-polyphosphate in their diet. Aquac. Int..

[32] Kanazawa A. (1995). Recent developments in shrimp nutrition and feed industry. INDAQUA ’95 Exposition of Indian Aquaculture, Madras, 27–30 January 1995.

[33] Radhakrishnan S., Bhavan P.S., Seenivasan C., Shanthi R., Muralisankar T. (2014). Replacement of fishmeal with *Spirulina platensis*, *Chlorella vulgaris* and *Azolla pinnata* on non-enzymatic and enzymatic antioxidant activities of *Macrobrachium rosenbergii*. J. Basic Appl. Zool..

[34] Tayag C.M., Lin Y.C., Li C.C., Liou C.H., Chen J.C. (2010). Administration of the hot-water extract of *Spirulina platensis* enhanced the immune response of white shrimp *Litopenaeus vannamei* and its resistance against *Vibrio alginolyticus*. Fish Shellfish Immunol..

[35] Newaj-Fyzul A., Austin B. (2015). Probiotics, immunostimulants, plant products and oral vaccines, and their role as feed supplements in the control of bacterial fish diseases. J. Fish Dis..

[36] Debtanu Barman P.N., Mandal S.C., Kumar V. (2013). Immunostimulants for Aquaculture Health Management. J. Mar. Sci. Res. Dev..

[37] Carton-Kawagoshi R.J., Caipang C.M., Caipang C.M., Bacano-Maningas M.B., Fagutao F.F. (2015). Algal-derived products and their role in shrimp immunity. Biotechnological Advances in Shrimp Health Management in the Philippines.

[38] Wang H., Dai A., Liu F., Guan Y. (2015). Effects of dietary astaxanthin on the immune response, resistance to white spot syndrome virus and transcription of antioxidant enzyme genes in Pacific white shrimp *Litopenaeus vannamei*. Iran. J. Fish. Sci..

[39] Supamattaya K., Kiriratnikom S., Boonyaratpalin M., Borowitzka L. (2005). Effect of a *Dunaliella* extract on growth performance, health condition, immune response and disease resistance in black tiger shrimp (*Penaeus monodon*). Aquaculture.

[40] Madhumathi M., Rengasamy R. (2011). Antioxidant status of *Penaeus monodon* fed with *Dunaliella salina* supplemented diet and resistance against WSSV. Int. J. Eng. Sci. Technol..

[41] Doron L., Segal N.A., Shapira M. (2016). Transgene expression in microalgae—From tools to applications. Front. Plant Sci..

[42] Yan N., Fan C., Chen Y., Hu Z. (2016). The potential for microalgae as bioreactors to produce pharmaceuticals. Int. J. Mol. Sci..

[43] Scaife M.A., Nguyen G.T.D.T., Rico J., Lambert D., Helliwell K.E., Smith A.G. (2015). Establishing *Chlamydomonas reinhardtii* as an industrial biotechnology host. Plant J..

[44] Dyo Y.M., Purton S. (2018). The algal chloroplast as a synthetic biology platform for production of therapeutic proteins. Microbiology.

[45] Economou C., Wannathong T., Szaub J., Purton S. (2014). A simple, low-cost method for chloroplast transformation of the green alga *Chlamydomonas reinhardtii*. Methods Mol. Biol..

[46] Rathod J.P., Gade R.M., Rathod D.R., Dudhare M. (2017). A review on molecular tools of microalgal genetic transformation and their application for overexpression of different genes. Int. J. Curr. Microbiol. App. Sci..

[47] Jeon S., Lim J., Lee H., Shin S., Kang N., Park Y.I., Oh H.-M., Jeong W.-J., Jeong B., Chang Y.K. (2017). Current status and perspectives of genome editing technology for microalgae. Biotechnol. Biofuels.

[48] Wannathong T., Waterhouse J.C., Young R.E., Economou C.K., Purton S. (2016). New tools for chloroplast genetic engineering allow the synthesis of human growth hormone in the green alga *Chlamydomonas reinhardtii*. Appl. Microbiol. Biotechnol..

[49] Feng S., Li X., Xu Z., Qi J. (2014). *Dunaliella salina* as a novel host for the production of recombinant proteins. Appl. Microbiol. Biotechnol..

[50] Scranton M.A., Ostrand J.T., Georgianna D.R., Lofgren S.M., Li D., Ellis R.C., Carruthers D.N., Dräger A., Masica D.L., Mayfield S.P. (2016). Synthetic promoters capable of driving robust nuclear gene expression in the green alga *Chlamydomonas reinhardtii*. Algal Res..

[51] Rasala B.A., Muto M., Sullivan J., Mayfield S.P. (2011). Improved heterologous protein expression in the chloroplast of *Chlamydomonas reinhardtii* through promoter and 5′ untranslated region optimization. Plant Biotechnol. J..

[52] Li S.S., Tsai H.J. (2009). Transgenic microalgae as a non-antibiotic bactericide producer to defend against bacterial pathogen infection in the fish digestive tract. Fish Shellfish Immunol..

[53] Feng S., Feng W., Zhao L., Gu H., Li Q., Shi K., Guo S., Zhang N. (2014). Preparation of transgenic *Dunaliella salina* for immunization against white spot syndrome virus in crayfish. Arch. Virol..

[54] Unajak S., Kiataramgul A., Wannathong T.W., Mavichak R., Yokthongwattana C., Areechon N. Development of *Chlamydomonas reinhardtii* for control white spot syndrome virus in shrimp (*Penaeus vannamei*). Proceedings of the Asian-Pacific Aquaculture 2016 Conference.

[55] Siripornadulsil S., Dabrowski K., Sayre R. (2007). Microalgal vaccines. Adv. Exp. Med. Biol..

[56] Surzycki R., Greenham K., Kitayama K., Dibal F., Wagner R., Rochaix J.D., Ajam T., Surzycki S. (2009). Factors effecting expression of vaccines in microalgae. Biologicals.

[57] Goldschmidt-Clermont M. Fischimpfung mit Chlamydomonas, die Bakterielle Antigene im Chloroplast Exprimieren. https://tinyurl.com/ydhzydbu.

[58] Michelet L., Lefebvre-Legendre L., Burr S.E., Rochaix J.D., Goldschmidt-Clermont M. (2011). Enhanced chloroplast transgene expression in a nuclear mutant of *Chlamydomonas*. Plant Biotechnol. J..

[59] Somchai P., Jitrakorn S., Thitamadee S., Meetam M., Saksmerprome V. (2016). Use of microalgae *Chlamydomonas reinhardtii* for production of double-stranded RNA against shrimp virus. Aquac. Rep..

[60] Saksmerprome V., Charoonnart P., Gangnonngiw W., Withyachumnarnkul B. (2009). A novel and inexpensive application of RNAi technology to protect shrimp from viral disease. J. Virol. Methods.

[61] Thammasorn T., Sangsuriya P., Meemetta W., Senapin S., Jitrakorn S., Rattanarojpong T., Saksmerprome V. (2015). Large-scale production and antiviral efficacy of multi-target double-stranded RNA for the prevention of white spot syndrome virus (WSSV) in shrimp. BMC Biotechnol..

[62] Saksmerprome V., Thammasorn T., Jitrakorn S., Wongtripop S., Borwornpinyo S., Withyachumnarnkul B. (2013). Using double-stranded RNA for the control of Laem-Singh Virus (LSNV) in Thai P. monodon. J. Biotechnol..

[63] Kumar A., Wang S., Ou R., Samrakandi M., Beerntsen B.T., Sayre R.T. (2013). Development of an RNAi based microalgal larvicide to control mosquitoes. Malar. World J..

[64] Gumpel N.J., Purton S. (1994). Playing tag with *Chlamydomonas*. Trends Cell Biol..

[65] Bertalan I., Munder M.C., Weiß C., Kopf J., Fischer D., Johanningmeier U. (2015). A rapid, modular and marker-free chloroplast expression system for the green alga *Chlamydomonas reinhardtii*. J. Biotechnol..

[66] Charoonnart P. (2018). National Center for Genetic Engineering and Biotechnology, National Science and Technology Development Agency, Thailand.

[67] Scaife M.A., Smith A.G. (2016). Towards developing algal synthetic biology. Biochem. Soc. Trans..

[68] Stevens D.R., Purton S. (1997). Genetic engineering of eukaryotic algae: Progress and prospects. J. Phycol..

[69] Ferenczi A., Pyott D.E., Xipnitou A., Molnar A. (2017). Efficient targeted DNA editing and replacement in *Chlamydomonas reinhardtii* using Cpf1 ribonucleoproteins and single-stranded DNA. Proc. Natl. Acad. Sci. USA.

[70] Slattery S.S., Diamond A., Wang H., Therrien J.A., Lant J.T., Jazey T., Lee K., Klassen Z., Desgagné-Penix I., Karas B.J. (2018). An expanded plasmid-based genetic toolbox enables Cas9 genome editing and stable maintenance of synthetic pathways in *Phaeodactylum tricornutum*. ACS Synth. Biol..

[71] Huang W., Daboussi F. (2017). Genetic and metabolic engineering in diatoms. Philos. Trans. R. Soc. Lond. B Biol. Sci..

[72] Potvin G., Zhang Z. (2010). Strategies for high-level recombinant protein expression in transgenic microalgae: A review. Biotechnol. Adv..

[73] Seo J., Chung H., Kim T. (2013). Codon-optimized expression of fish iridovirus capsid protein in yeast and its application as an oral vaccine candidate. J. Fish Dis..

[74] Fujiwara T., Kanesaki Y., Hirooka S., Era A., Sumiya N., Yoshikawa H., Tanaka K., Miyagishima S.Y. (2015). A nitrogen source-dependent inducible and repressible gene expression system in the red alga *Cyanidioschyzon merolae*. Front. Plant Sci..

[75] Iwai M., Hori K., Sasaki-Sekimoto Y., Shimojima M., Ohta H. (2015). Manipulation of oil synthesis in *Nannochloropsis* strain NIES-2145 with a phosphorus starvation-inducible promoter from *Chlamydomonas reinhardtii*. Front. Microbiol..

[76] Stoffels L., Taunt H.N., Charalambous B., Purton S. (2017). Synthesis of bacteriophage lytic proteins against *Streptococcus pneumoniae* in the chloroplast of *Chlamydomonas reinhardtii*. Plant Biotechnol. J..

[77] Kalatzis P.G., Castillo D., Katharios P., Middelboe M. (2018). Bacteriophage interactions with marine pathogenic Vibrios: Implications for phage therapy. Antibiotics.

[78] Chen H.L., Li S.S., Huang R., Tsai H.J. (2008). Conditional production of a functional fish growth hormone in the transgenic line of *Nannochloropsis oculata* (Eustigmatophyceae). J. Phycol..

[79] Kim D.H., Kim Y.T., Cho J.J., Bae J.H., Hur S.B., Hwang I., Choi T.J. (2002). Stable integration and functional expression of flounder growth hormone gene in transformed microalga, *Chlorella ellipsoidea*. Mar. Biotechnol..

[80] Shin Y., Kwon T., Seo J., Kim T. (2013). Oral immunization of fish against iridovirus infection using recombinant antigen produced from rice callus. Vaccine.

[81] Allnutt F.C., Bowers R.M., Rowe C.G., Vakharia V.N., LaPatra S.E., Dhar A.K. (2007). Antigenicity of infectious pancreatic necrosis virus VP2 subviral particles expressed in yeast. Vaccine.

[82] Norsker N.H., Barbosa M.J., Vermuë M.H., Wijffels R.H. (2011). Microalgal production—A close look at the economics. Biotechnol. Adv..

[83] Acién F.G., Fernández J.M., Magán J.J., Molina E. (2012). Production cost of a real microalgae production plant and strategies to reduce it. Biotechnol. Adv..

[84] Xu Y., Purton S., Baganz F. (2013). Chitosan flocculation to aid the harvesting of the microalga *Chlorella sorokiniana*. Bioresour. Technol..

[85] Ahmed F., Li Y., Fanning K., Netzel M., Schenk P.M. (2015). Effect of drying, storage temperature and air exposure on astaxanthin stability from *Haematococcus pluvialis*. Food Res. Int..

[86] Liu B., Yu Z., Song X., Guan Y. (2007). Studies on the transmission of WSSV (white spot syndrome virus) in juvenile *Marsupenaeus japonicus* via marine microalgae. J. Invertebr. Pathol..

[87] The Convention on Biological Diversity Nagoya Protocol. https://www.cbd.int/abs/about/.

[88] Gressel J., van der Vlugt C.J.B., Bergman H.E.N. (2013). Environmental risks of large scale cultivation of microalgae: Mitigation of spills. Algal Res..

[89] Glass D.J., Prokop A., Bajpai R., Zappi M. (2015). Government regulation of the uses of genetically modified algae and other microorganisms in biofuel and bio-based chemical production. Algal Biorefineries.

[90] Henley W., Litaker W., Novoveská L., Duke C., Quemada H., Sayre R.T. (2013). Initial risk assessment of genetically modified (GM) algae for commodity-scale cultivation. Algal Res..

[91] Fu J., Yang D., Jin M., Liu W., Zhao X., Li C., Zhao T., Wang J., Gao Z., Shen Z. (2017). Aquatic animals promote antibiotic resistance gene dissemination in water via conjugation: Role of different regions within the zebra fish intestinal tract, and impact on fish intestinal microbiota. Mol. Ecol..

[92] Young R.E.B., Purton S. (2016). Codon reassignment to facilitate genetic engineering and biocontainment in the chloroplast of *Chlamydomonas reinhardtii*. Plant Biotechnol. J..

[93] Lucht J.M. (2015). Public acceptance of plant biotechnology and GM crops. Viruses.

